# Profound remission in Crohn’s disease requiring no further treatment for 3–23 years: a case series

**DOI:** 10.1186/s13099-020-00355-8

**Published:** 2020-04-09

**Authors:** Gaurav Agrawal, Annabel Clancy, Roy Huynh, Thomas Borody

**Affiliations:** Centre for Digestive Diseases, 1/299 Great North Road, Five Dock, NSW 2046 Australia

**Keywords:** Crohn disease, Anti-bacterial therapy, *Mycobacterium paratuberculosis*, Inflammatory bowel disease, Faecal microbiota transplant

## Abstract

**Background:**

Crohn’s disease (CD) is rising in incidence and has a high morbidity and increased mortality. Current treatment use immunosuppressives but efficacy is suboptimal, and relapse is common. It has been shown that there is an imbalance present in the gut microbiome (dysbiosis) in CD with a possible infective aetiology—*Mycobacterium avium* subsp. *paratuberculosis* (MAP) being the most proposed. Antibacterial therapy and Faecal Microbiota Transplantation (FMT) are emerging treatments which can result in clinical and endoscopic remission, if employed correctly. The objective of this study was to report on the treatment and clinical outcomes of patients with CD in prolonged remission.

**Results:**

Ten patients were identified to have achieved prolonged remission for 3–23 years (median 8.5 years). Of these, 7/10 took targeted Anti-MAP therapy (AMAT) for a median 36 months and then ceased AMAT treatment. After stopping AMAT five patients underwent Faecal Microbiota Transplantation (FMT) (average four infusions). In 4/7, AMAT was combined with infliximab (mean of six infusions) that was withdrawn within 6 months after fistulae resolution. One patient achieved deep mucosal healing with AMAT alone. Of the 3/10 patients not prescribed AMAT, one had a combination of anti-inflammatory agents and a single antibiotic (metronidazole) followed by FMT. The other two received only FMT for *Clostridioides difficile* Infection.

**Conclusions:**

Prolonged remission has been achieved for 3–23 years with individualised treatments, with the majority using AMAT ± infliximab and FMT. Treatment with antibiotics and/or FMT provides a potential new avenue for treatment of CD. These findings should stimulate thinking, investigations and better therapy against MAP and the dysbiosis of the gut flora, to enable higher rates of prolonged remission.

## Background

Crohn’s disease (CD) is a chronic inflammatory process of the digestive tract characterized by deep ulcerations, skip lesions, transmural inflammation, fistulae and granulomas, with no known cure. It has a negative impact on many aspects of quality of life, including physical, social, psychological, and sexual functioning. Most patients are diagnosed in the second or third decade of life, during their most productive years. CD is associated with significant morbidity, including frequent emergency room admissions, and surgical interventions. There is also an increased mortality, primarily due to complications associated with CD. Mucosal healing, or ‘endoscopic remission’, is associated with clinical remission, fewer hospitalizations and abdominal surgeries [1].

For decades, anti-inflammatory and immunosuppressive agents, designed to dampen down the immune response, have been the standard of care in CD [1]. With these traditional therapies, the goal is control of inflammation to induce clinical remission. More recently, biologics targeted towards various cytokines and components of the inflammatory cascade have been approved for CD [2]. With the advent of disease-modifying therapies, new and increasingly ambitious treatment goals have been sought, ranging from mere control of clinical symptoms towards mucosal healing and “deep remission,” a term introduced to encompass mucosal healing plus clinical and/or laboratory improvement [3]. However, despite initial promise of such drugs, more than 60% of patients fail to respond to biologics by 6 weeks, and roughly 50% fail to achieve remission by 1 year [2]. A further 23–46% of patients experience a loss of response over time. Despite significant expenditure on biologics [4], and more than a century of existence of CD as a clinical entity, we are no closer to finding a cure for CD, which confirms that merely damping the immune response is inadequate to achieve this.

An association between *Mycobacterium avium* subsp. *paratuberculosis* (MAP) and CD has been proposed as early as 1913, given striking clinical and histological similarities to inflammatory bowel disease of ruminants; “Johne’s disease”, as well as ileo-caecal tuberculosis in humans [5]. Both caused from Mycobacteria, with the former being MAP. Following these observations, conflict has continued as to its true role. Data from epidemiological [6–8], genetic [9, 10], microbiologic, experimental human and animal studies [11], clinical trials [12–14], and meta-analyses [15, 16], have led to the support for a pathogenic role for MAP in CD. Strengthening this argument, MAP has been cultured from a patient with CD and transmitted to goats, who developed non-caseating ileal granulomas from which the same pathogen was again cultured [11, 17]. However, given the inherent difficulties in culturing and detecting this agent consistently [16], as well as the negative studies that have failed to find a link between MAP and CD [18], and the lack of a specific diagnostic method, a role for MAP in CD is controversial.

The efficacy of antibiotics targeted against MAP; Anti-MAP therapy or AMAT, has now been demonstrated in several prospective trials, with clinical remission rates approaching 93% [3, 6, 7]. In the first large-scale, randomized, controlled trial of AMAT in CD, an “intention-to-treat” re-analysis (correcting for statistical errors), showed that AMAT achieved significant induction of remission as well as maintenance of remission [19]. A subsequent international, Phase III Randomised Control Trial (RCT) using a fixed dose AMAT in 331 CD patients, met its primary endpoint of clinical remission, defined as Crohn’s Disease Activity Index (CDAI) < 150 by week 26. Key secondary endpoints, including clinical response at week 26 (p = 0.016), early clinical remission at week 16 (p = 0.015), clinical remission at week 16 and 52 (p = 0.003) and durable remission at all visits through week 52 (p = 0.018) were also met [13]. Despite these, and earlier observations documenting complete mucosal healing with AMAT [20, 21], there is no published evidence of prolonged remission and mucosal healing in the absence of ongoing therapy. Here, we report a subset of CD patients treated in an individualised manner, with the majority using AMAT.

Furthermore, it has been shown that there is an imbalance present in the gut microbiome (dysbiosis) in CD. This is composed of an abundance of detrimental bacterial species, such as Enterobacteriaceae (facultative anaerobic bacteria) and a corresponding reduction of beneficial species such as Bacteroides, and firmicutes [22]. Faecal Microbiota Transplantation (FMT), the introduction of healthy human donor stool into the bowel of the recipient, has been suggested as a possible ideal therapeutic agent to restore gut flora imbalances in CD [23]. To date, small studies have shown inconsistent results of FMT in CD and the therapeutic effects are less than that seen in Ulcerative Colitis or *Clostridioides difficile* infection, for reasons currently unknown [24]. However, one trial revealed an improvement in 76% of patients, 1 year after a single infusion of FMT [25]. Here, we also report a small subset of CD patients treated with FMT.

Antibacterial therapy and FMT are emerging treatments which can result in clinical and endoscopic remission. However, evidence of prolonged remission has not yet been established but in our experience with patients treated with AMAT, approximately 70% of patients are induced into remission (Collyer et al. Under Review). FMT has further maintained this remission, enabling reduction of medications. The objective of this study was to report on the treatment and clinical outcomes of patients with CD in prolonged remission.

## Results

**Fig. 1:**
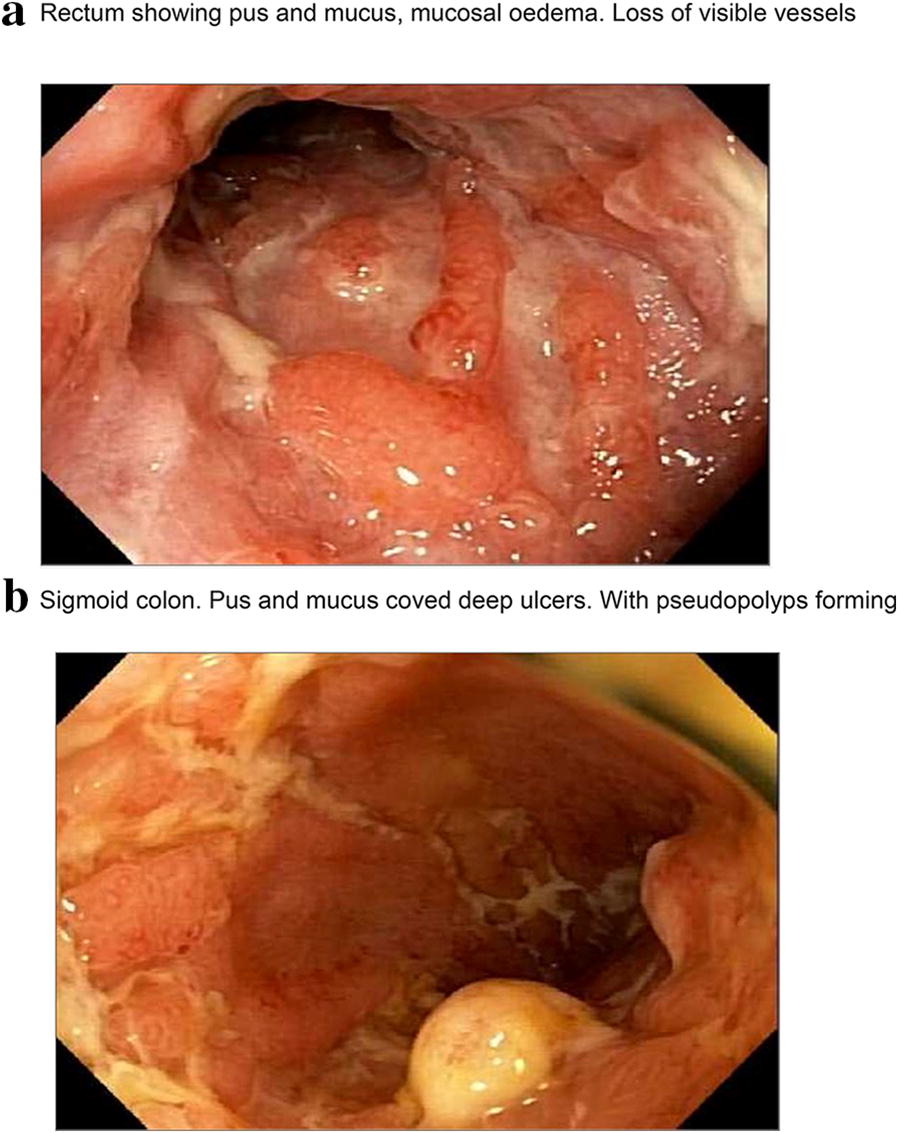
Patient 7: Before anti-*Mycobacterium avium paratuberculosis* therapy (AMAT). **a** Rectum showing pus and mucus, mucosal oedema. Loss of visible vessels. **b** Sigmoid colon. Pus and mucus coved deep ulcers. With pseudopolyps forming

**Fig. 2:**
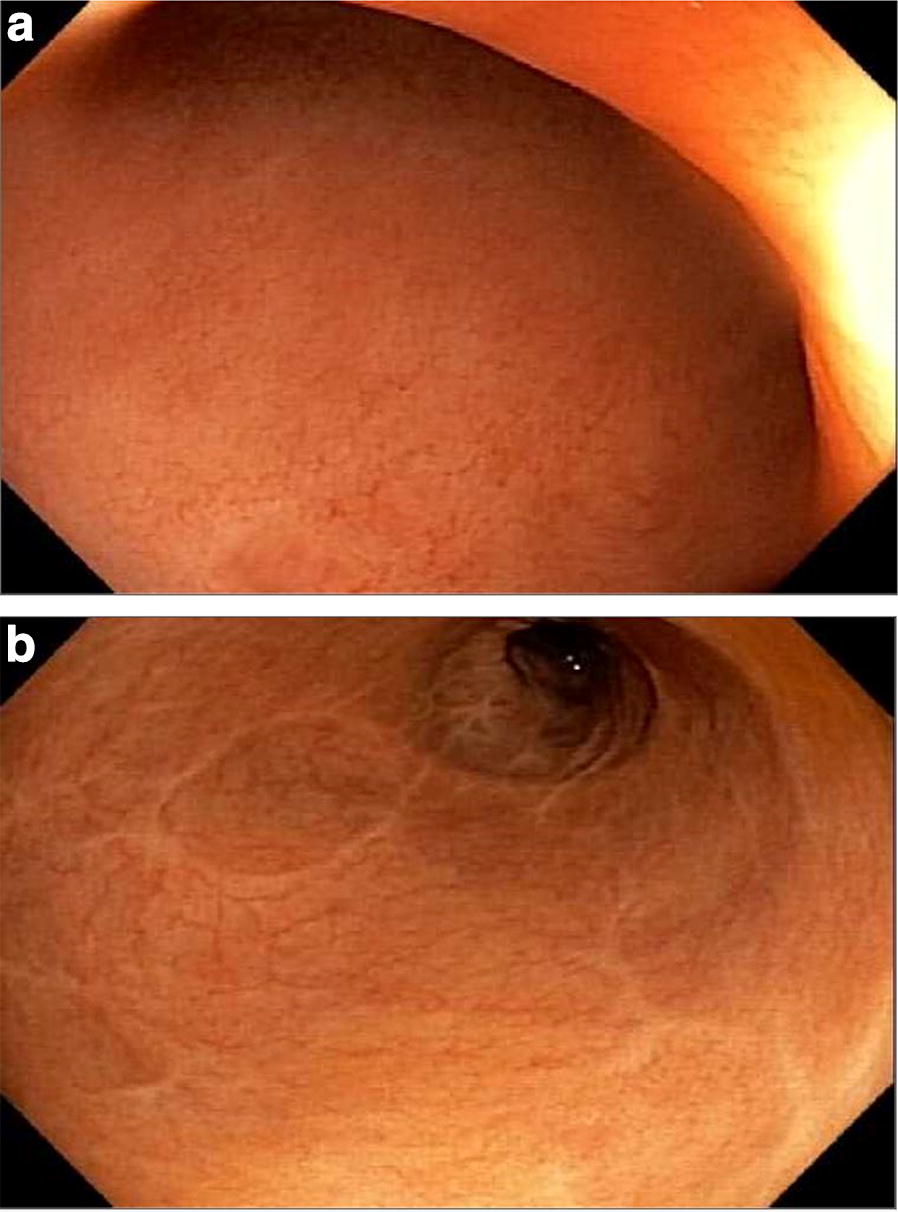
Patient 7: Follow-up 18 months after AMAT. **a** Rectum. Pattern of fine network of scars showing where ulcers had healed. No inflammation. **b** Sigmoid colon

**Fig. 3:**
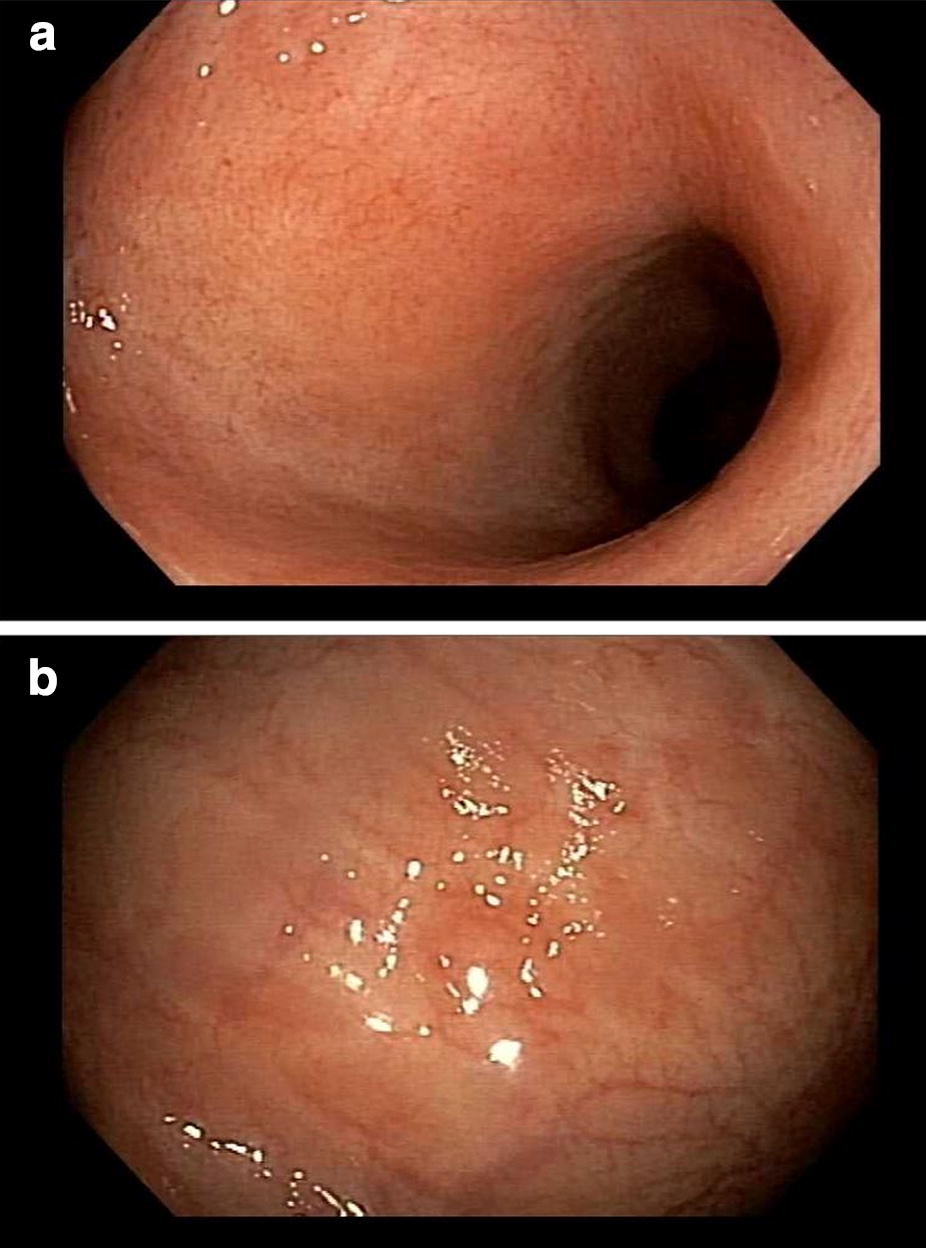
Patient 7: Follow up 24 months after Faceal Microbiota Transplant. **a** Rectum. **b** Sigmoid colon

Approximately 350 patients with CD were seen at our clinic from November 2016 to November 2018. Follow-up is usually annual for all patients in remission. A total of 10 patients (16–56 years; 7F:3 M) were identified to meet the inclusion criteria. All 10 patients were clinically asymptomatic with a CDAI of < 30. They had endoscopic resolution as well as histologically complete, deep mucosal healing (Figs. 1, 2 and 3) and had ceased all CD treatments. Median duration of treatment free remission was 8.5 years (3–23 years).

Of these patients, seven had been treated with AMAT regimens comprising 3–5 antibiotics, including rifabutin, clarithromycin, clofazimine, and in some metronidazole, ciprofloxacin or pyrazinamide (Table 1). In addition, all subjects had Interferon gamma negative tests to rule out *Mycobacterium tuberculosis* (MTB). AMAT was administered using a dose-escalation over an average of 8 weeks to reach final doses of rifabutin (4 mg/kg), clofazimine (3.75 mg/kg) and clarithromycin (14 mg/kg). Median time to remission was 7 months (range of 6–12 months). The combination antibiotic therapy was personalised according to factors, such as allergies, fistulae, severity of presentation and initial response to three antibiotics. In four patients, AMAT was combined with infliximab (mean 6 infusions) to accelerate fistula healing, as shown in one analysis using combination therapy [26]. One patient achieved deep mucosal healing with AMAT alone. Following cessation of AMAT (median of 36 months of treatment), five patients underwent FMT (average of four infusions). Donors were single sourced and had been screened for known pathogens via blood and stool tests, as well as family and personal clinical history.

**Table 1:** Characteristics of patients achieving prolonged treatment-free remission and endoscopic healing following treatment (N = 10)

Patient	Age, years	Sex	Prior therapies	AMAT (Y/N)	AMAT Regimen	Duration of AMAT (years)	Adjunct therapy	Duration of treatment-free remission (years)
1	47	M	Azathioprine^a^	N	–	–	FMT methotrexate vitamin D	4
2	21	M	Prednisone, right hemi-colectomy	Y	Rifabutin; clofazimine; clarithromycin, Ciprofloxacin, metronidazole	1	Anti-TNF infliximab infusion, vitamin D	9
3	56	F	Sulfasalazine; mesalazine^a^; azathioprine^a^; esomeprazole; Metronidazole^a^; Vancomycin^a^.	N	–	–	FMT	6
4	43	M	Azathioprine^a^; Mesalazine^a^; Esomeprazole.	N	–	–	FMT	23
5	17	F	Ciprofloxacin^a^, metronidazole^a^, azathioprine^a^; Prednisone	Y	Rifabutin; clofazimine; clarithromycin, ciprofloxacin, metronidazole and ethambutol	9	Infliximab	18
6	48	F	Mesalazine^a^	Y	Rifabutin; clofazimine; clarithromycin, ethambutol	3	FMT	14
7	36	F	Adalimumab; Infliximab (alone); azathioprine^a^; ciprofloxacin^a^; metronidazole^a^	Y	Rifabutin; clofazimine; clarithromycin; metronidazole	2	FMT	3
8	38	F	Mesalazine^a^	Y	Rifabutin; clofazimine; clarithromycin, metronidazole, ciprofloxacin	8	Infliximab	8
9	32	F	Prednisone; metronidazole^a^; ciprofloxacin^a^	Y	Rifabutin; clofazimine; clarithromycin, metronidazole, ciprofloxacin	3	–	8
10	16	F	Mesalazine^a^; azathioprine^a^	Y	Rifabutin; clofazimine; clarithromycin, ethambutol	8	Infliximab	11

*AMAT* anti-mycobacterial antibiotic therapy, *FMT* faecal microbiota transplantation

^a^Therapies known to have potential anti-MAP properties

Of the patients not prescribed AMAT (n = 3), one (patient #3) used a combination of anti-inflammatory therapy (azathioprine and 5-ASA) and antibiotics (metronidazole), followed by six FMTs, to achieve prolonged healing. The remaining two patients underwent single FMT via colonoscopic infusion for treatment of *Clostridioides difficile* Infection.

Of note, many other patients were identified to be in long-term remission but had been unable to completely cease medication without relapse, which could have been months or years later. They would typically be on lower dose ‘maintenance’ AMAT or 5-ASA (mesalamine) compounds to maintain remission.

## Discussion

Deep mucosal healing in CD has been reported previously with CD treatments, including infliximab, azathioprine, methotrexate [27], but not seen with 5-aminosalicylates and corticosteroids [28]. In a small sub study conducted by D’Haens et al. (2002) of 19 patients who completed the ACCENT-1 trial, the effect of scheduled (every 8 weeks) versus on demand infliximab treatment on mucosal healing was examined [29]. The median time to relapse was 12 weeks (range 1- > 78 weeks). In the EXTEND trial, which evaluated the efficacy of adalimumab for 52 weeks in moderate-to-severe CD, 27% of patients receiving adalimumab had mucosal healing at week 12 (the primary end point) versus 13% given placebo (p = 0.056). At week 52, rates of mucosal healing were 24% and 0, respectively (p < 0.001) [30]. In a study examining the effects of mucosal healing on long-term outcomes in CD, 64.8% of patients who achieved mucosal healing were in clinical remission at the end of 5 years, whereas only 39.5% of patients who did not achieve mucosal healing attained clinical remission [31]. A pertinent observation from this is that the patients in the trials described above were unable to cease CD therapy without relapsing. There is no long-term follow up data past 2 years.

The treatments described in this case series were individualised to the patient. Most patients (70%) had received AMAT for a period of 3 years to initially induce deep healing, which translated clinically to a remission of their disease. AMAT consisted of a combination of agents effective against MAP, some patients requiring addition of alternate AMAT agents due to occasional adverse effects, generally being arthralgia. The length of treatment was based on observations of *Mycobacterium leprae* treatment (12–18 months) and that MAP is an even slower growing/reproducing mycobacteria. Several patients received FMT to re-establish a healthy gut microbiome after prolonged antibiotic use. In addition, two patients achieved long-term clinical remission and mucosal healing with FMT alone. This adds further support to an infective aetiology of CD because FMT eradicates pathogens, for example *Clostridioides difficile* infection, and may have a parallel action in CD. The important observation is that long-term remission here was achieved, not via immune modulating biologics targeting inflammation but rather by the use of antibiotics targeted against MAP and/or FMT targeting dysbiosis of the gut flora. Further optimally designed trials comparing therapy with AMAT and FMT versus current ‘standard of care’ therapy in CD should be conducted. Long-term follow up studies of patients with CD treated with AMAT and/or microbiome modification are also required to confirm our results.

These observations concur with our notion that CD occurs as the result of a primary infection with MAP, or a similar pathogen, and a resulting disruption of the protective mechanisms of the gut microbiome (dysbiosis). We hypothesize that there is a complex interplay, where a predisposition, stemming from innate immune deficiencies (given this particularly is involved in intracellular bacterial infections and perhaps evolved alongside the resident gut flora), that likely arise from certain deficiencies of the gut microbiome. This has been shown to be the case in tuberculosis susceptibility [32]. Genetic factors, such as the NOD2CARD15 mutations contributes to risk of developing disease, as these enable the pathogenesis of CD and also have been shown to confer an increased risk to mycobacterial infections [33, 34]. MAP itself can further disrupt the biodiversity of the microbial environment of the gut [35], leading to excessive and maladaptive immune responses, which current immune suppressive therapy target. MAP behaviour is similar to other mycobacteria, where there are high infectivity rates but low expression/pathogenicity. The rates are in part given to the respective reproductive rates, with MAP having one of the longest known of the Mycobacteria *genus*. *Mycobacterium tuberculosis* (MTB) infects a third of the world’s population (~ 3 billion), yet disease presents in 3-5% of individuals (WHO Global tuberculosis report 2015). However, one difference between MAP and the MTB complex is that MAP doesn’t lead to deterioration when immunosuppressives are given, just one of the different characteristics between atypical (*avium*) mycobacteria and MTB complex. A proposition, based on multiple studies, is that immunosuppressives actually work in CD by their secondary antibiotic action on MAP [36–38]. Interestingly, anti-TNF α agents have been shown to reduce Mycobacterial survivability and may be a key reason why they work well with AMAT to accelerate healing [39], especially that of Crohn's fistulae[26]. Though a caveat to this is that they may lead to resistance of MAP, where it resides in tissues [40]. A problem that we have observed in the response using AMAT in patients with a history of biologic use compared to ‘treatment-naïve’ patients [41].

The ability for MAP to influence the gut microbiome has been demonstrated in animal rabbit [35] and cattle [42] models. We propose that the inability to clear MAP from the macrophage and the resulting dysbiosis from viable mycobacteria, leads to granulomas and the relapsing, remitting behaviour observed in CD. Targeting both steps is likely key to higher eradication and recovery rates and a reason as to why efficacy of FMT alone is currently sub-optimal in remission of CD [43]. However, there may be certain circumstances in a minority where recovery of the dysbiosis alone (by FMT only) may be sufficient in clearing/containing MAP, perhaps through restoration of the (innate) immune system functioning. In addition, the dysbiosis of the gut microbiome is a likely reason why dietary intervention plays a key role in the symptoms and treatment of CD [44], by altering (± the activity) of the gut microbiota and so modulating inflammation. Addressing this step after the primary treatment with AMAT may also be critical for long-term remission and requires further evaluation. In addition, sensitivities of MAP to the antibiotic chemotherapy (which are predominately intracellular acting compounds, particularly for Mycobacteria e.g. clofazimine), coupled with the ability to implant healthy commensal microbes will also likely influence the success rates. MAP, like other mycobacteria, has been shown to have the presence of resistant strains to Rifamycin compounds (including Rifampicin and Rifabutin), due to the presence of the rpoB gene. This will influence the response to AMAT [45–47].

In addition, further research into the gut dysbiosis, including the role of specific pathogens such as MAP, is required to improve understanding and improving treatment efficacy in CD.

Case series have the benefit of reporting novel clinical outcomes that cannot be established from short-term, expensive RCT research protocols. They in essence highlight principles of a treatment. However, the inherent limitations of case reports including; small sample size, single centre source and clinical nature of the information, resulting in missing data, are recognised and limits the consistency/reproducibility of the results. It is acknowledged that there could be a wide range of possible variables, which could have influenced the outcomes seen. However, this is a precursor highlighting the requirement of larger studies, which will enable multi-variate statistical testing based on a more complete dataset, including negative outcomes, to be utilised. However, given that currently it is thought Crohn’s is incurable with immunosuppressive treatments, alternative lines of causation and treatment should also be investigated in parallel.

A significant limitation of this study is the lack of MAP testing in patients. The unavailability of culturing, cataloguing and diagnostic tests of the microbiome and MAP during this period, has hindered the details and understanding of these results and effects, for which these patients were treated for. Improvements for future research will require better diagnostics for MAP, cataloguing of the gut microbiome before and after therapy, as well as standardised clinical, endoscopic and histological measures in prospective or randomised methods. However, it is likely that standardised treatment regimens may never be achieved. The main factors influencing outcomes are individual responses and immune functioning, use of prior biologics (which appears to make MAP more resistant), and the resistant nature of the organism that is currently seen and understood. This is a pattern reminiscent in *Mycobacterium avium* and *tuberculosis* therapy, where Severe-Drug resistant TB (XDR-TB) eradication rate is 35% compared to fully sensitive MTB of 81% (WHO Global Health report). Influencing FMT factors will include implantation success, donor suitability and number of infusions.

## Conclusion

In conclusion, we report sustained remission of CD for 3–23 years in a group of patients after targeting MAP and the gut microbiome. Treatment with antibiotics and/or FMT provides a potential new avenue for treatment of CD. Randomised controlled trials comparing therapy with AMAT and FMT versus current ‘standard of care’ therapy in CD are required. These findings should stimulate thinking, investigations and better therapy against the dysbiosis of the gut flora, to enable higher rates of prolonged remission.

## Methods

This was a single centre, retrospective review of patients with CD attending our clinic between November 2016 and November 2018. Patients with a history of confirmed CD diagnosis from clinical, endoscopic and histopathological analysis and identified to be in prolonged remission were included. This was defined as a normal CDAI (< 150), endoscopic appearance, absent inflammation on histology and clinically asymptomatic for a period of greater than 3 years without any CD therapies. This figure of ‘3 years’ was chosen from our clinical observations that after total healing and off all therapy those who still had CD would have relapsed before this time point. Patients identified had their medical records tabulated in November 2018 to include demographic data, symptoms, endoscopic and histological findings, and treatment regimens and durations. Deep mucosal healing was deemed present as per Rogler et al. 2013 [3]. Duration of treatment-free remission was defined as continuous period of time off all active CD treatments. A second, independent reviewer, to ensure accuracy, checked the data. Descriptive statistics were conducted using Microsoft Excel.

## Data Availability

There is no associated data set for this manuscript.

## References

[1] Lichtenstein GR, Loftus EV, Isaacs KL (2018). ACG clinical guideline: management of Crohn’s Disease in Adults. Am J Gastroenterol.

[2] Feagan BG, Sandborn WJ, Gasink C (2016). Ustekinumab as induction and maintenance therapy for Crohn’s Disease. N Engl J Med.

[3] Rogler G, Vavricka S, Schoepfer A (2013). Mucosal healing and deep remission: what does it mean?. World J Gastroenterol.

[4] Yu H, Maclsaac D, Wong J (2018). Market share and costs of biologic therapies for inflammatory bowel disease in the USA. Aliment Pharmacol Ther.

[5] Davis WC, Kuenstner JT, Singh SV (2017). Resolution of Crohn’s (Johne’s) disease with antibiotics: what are the next steps?. Expert Rev Gastroenterol Hepatol..

[6] Green C, Elliott L, Beaudoin C (2006). A population-based ecologic study of inflammatory bowel disease: searching for etiologic clues. Am J Epidemiol..

[7] Pickup R, Rhodes G, Arnott S (2005). *Mycobacterium avium* subsp. paratuberculosis in the catchment area and water of the river Taff in South Wales, United Kingdom, and its potential relationship to clustering of Crohn’s Disease Cases in the City of Cradiff. Appl Environ Microbiol.

[8] Gearry RB, Chb MB, Richardson A (2006). High incidence of Crohn’s disease in Canterbury, New Zealand: results of an epidemiologic study. Inflamm Bowel Dis.

[9] Franke A, McGovern DPB, Barrett JC (2010). Genome-wide meta-analysis increases to 71 the number of confirmed Crohn’s disease susceptibility loci. Nat Genet.

[10] Strober W, Asano N, Fuss I (2014). Cellular and molecular mechanisms underlying NOD2 risk-associated polymorphisms in Crohn’s disease. Immunol Rev.

[11] Van Kruiningen H, Chiodini R, Thayer W (1986). Experimental disease in infant goats induced by a *Mycobacterium* isolated from a patient with Crohn’s disease. Dig Dis Sci.

[12] Selby W, Pavli P, Crotty B (2007). Two-year combination antibiotic therapy with clarithromycin, rifabutin, and clofazimine for Crohn’s disease. Gastroenterology.

[13] Graham D (2018). Phase III randomised, double blind, placebo-controlled, multicenter, paralell group study to assess the efficacy and safety of add-on fixed-dose anti-mycobacterial therapy (RHB-104) in moderately to severely active Crohn’s disease. United Eur Gastroenterol..

[14] Gui GPH, Thomas PRS, Tizard MLV (1997). Two-year-outcomes analysis of Crohn’s disease treated with rifabutin and macrolide antibiotics. J Antimicrob Chemother.

[15] Abubakar I, Myhill D, Aliyu S (2008). Detection of MAP from patients with CD using nuclei acid-based techniques: a systematic review and meta-analysis. Inflamm Bowel Dis.

[16] Feller M, Huwiler K, Stephan R (2007). *Mycobacterium avium* subspecies paratuberculosis and Crohn’s disease: a systematic review and meta-analysis. Lancet Infect Dis..

[17] Blevins SM, Bronze MS (2010). Robert Koch and the “golden age” of bacteriology. Int J Infect Dis..

[18] Hermon-Taylor J (2001). *Mycobacterium avium* subspecies paratuberculosis is a cause of Crohn’s disease. Gut.

[19] Behr MA, Hanley J (2008). Reflection and reaction antimycobacterial therapy for Crohn’ s disease: a reanalysis. Lancet Infect Dis..

[20] Borody TJ, Bilkey S, Wettstein AR (2007). Anti-mycobacterial therapy in Crohn’s disease heals mucosa with longitudinal scars. Dig Liver Dis..

[21] Chamberlin W, Borody TJ, Campbell J (2011). Primary treatment of Crohn’s disease: combined antibiotics taking center stage. Expert Rev Clin Immunol..

[22] Joossens M, Huys G, Cnockaert M (2011). Dysbiosis of the faecal microbiota in patients with Crohn’s disease and their unaffected relatives. Gut.

[23] Xiang L, Ding X, Li Q (2020). Efficacy of faecal microbiota transplantation in Crohn’s disease: a new target treatment?. Microb Biotechnol.

[24] Borody TJ, Finlayson S, Paramsothy S (2014). Is Crohn’s disease ready for fecal microbiota transplantation?. J Clin Gastroenterol.

[25] Cui B, Feng Q, Wang H (2015). Fecal microbiota transplantation through mid-gut for refractory Crohn’s disease: safety, feasibility, and efficacy trial results. J Gastroenterol Hepatol.

[26] Agrawal G, Borody T, Turner R (2015). Combining infliximab, anti-MAP and hyperbaric oxygen therapy for resistant fistulizing Crohn’s disease. Futur Sci OA..

[27] Laharie D, Reffet A, Belleannée G (2011). Mucosal healing with methotrexate in Crohns disease: a prospective comparative study with azathioprine and infliximab. Aliment Pharmacol Ther.

[28] Kakkar A, Wasan S, Farraye F (2011). Targeting mucosal healing in Crohn’s disease. Gastroenterol Hepatol.

[29] D’Haens GR, Baert F (2002). Endoscopic healing after infliximab treatment for Crohn’s disease provides a longer time to relapse. Gastroenterology.

[30] Rutgeerts P, Van Assche G, Sandborn WJ (2012). Adalimumab induces and maintains mucosal healing in patients with Crohn’s disease: data from the EXTEND trial. Gastroenterology.

[31] Schnitzler F, Fidder H, Ferrante M (2009). Mucosal healing predicts long-term outcome of maintenance therapy with infliximab in Crohn’s disease. Inflamm Bowel Dis.

[32] Eribo OA, du Plessis N, Ozturk M (2019). The gut microbiome in tuberculosis susceptibility and treatment response: guilty or not guilty?. Cell Mol Life Sci.

[33] Economou M, Trikalinos TA, Loizou KT (2004). Differential effects of NOD2 variants on Crohn’s disease risk and phenotype in diverse populations: a metaanalysis. Am J Gastroenterol.

[34] Pan H, Dai Y, Tang S (2012). Polymorphisms of NOD2 and the risk of tuberculosis: a validation study in the Chinese population. Int J Immunogenet.

[35] Arrazuria R, Elguezabal N, Juste RA (2016). *Mycobacterium avium* subspecies paratuberculosis infection modifies gut microbiota under different dietary conditions in a rabbit model. Front Microbiol..

[36] Greenstein RJ, Su L, Shahidi A (2007). On the action of 5-amino-salicylic acid and sulfapyridine on *M. avium* including subspecies paratuberculosis. PLoS ONE.

[37] Greenstein RJ, Su L, Juste RA (2008). On the action of cyclosporine A, rapamycin and tacrolimus on *M avium* including subspecies paratuberculosis. PLoS ONE.

[38] Krishnan MY, Manning EJB, Collins MT (2009). Effects of interactions of antibacterial drugs with each other and with 6-mercaptopurine on in vitro growth of *Mycobacterium avium* subspecies paratuberculosis. J Antimicrob Chemother.

[39] Bach H, Rosenfeld H, Bressler B (2012). Treatment of Crohn’s disease patients with infliximab is detrimental for the survival of MAP. pdf. J Crohns Colitis..

[40] Qasem A, Naser SA (2018). TNFα inhibitors exacerbate *Mycobacterium paratuberculosis* infection in tissue culture: a rationale for poor response of patients with Crohn’s disease to current approved therapy. BMJ Open Gastroenterol.

[41] Agrawal G, Clancy A, Sharma R, Huynh R, Ramrakha S, Borody T (2020). Targeted combination antibiotic therapy induces remission in treatment-naïve Crohn’s disease: a case series. Microorganisms.

[42] Fecteau ME, Pitta DW, Vecchiarelli B (2016). Dysbiosis of the fecal microbiota in cattle infected with *Mycobacterium avium* subsp. *paratuberculosis*. PLoS One..

[43] Wang H, Cui B, Li Q (2018). The safety of fecal microbiota transplantation for Crohn’s disease: findings from a long-term study. Adv Ther..

[44] Rapozo DCM, Bernardazzi C, De Souza HSP (2017). Diet and microbiota in inflammatory bowel disease: the gut in disharmony. World J Gastroenterol.

[45] Beckler DR, Elwasila S, Ghobrial G (2008). Correlation between rpoB gene mutation in *Mycobacterium avium* subspecies *paratuberculosis* and clinical rifabutin and rifampicin resistance for treatment of Crohn’s disease. World J Gastroenterol.

[46] ​Zanetti S S, Molicotti P, Cannas S (2006). “In vitro” activities of antimycobacterial agents against *Mycobacterium avium* subsp. *paratuberculosis* linked to Crohn’s Disease and Paratuberculosis. Ann Clin Microbiol Antimicrob..

[47] ​Krishnan MY, ​Manning EJB, ​Collins MT (2009). Comparison of three methods for susceptibility testing of *Mycobacterium avium* subsp. *paratuberculosis* to 11 antimicrobial drugs. J Antimicrob Chemother..

